# Ultrasound-mediated disruption of the blood tumor barrier for improved therapeutic delivery

**DOI:** 10.1016/j.neo.2021.04.005

**Published:** 2021-06-14

**Authors:** T.A. Arsiwala, S.A. Sprowls, K.E. Blethen, C.E. Adkins, P.A. Saralkar, R.A. Fladeland, W. Pentz, A. Gabriele, B. Kielkowski, R.I. Mehta, P. Wang, J.S. Carpenter, M. Ranjan, U. Najib, A.R. Rezai, P.R. Lockman

**Affiliations:** a Department of Pharmaceutical Sciences, School of Pharmacy, West Virginia University, HSC, Morgantown, WV; b School of Pharmacy, South University, Savannah, GA; c Rockefeller Neuroscience Institute, West Virginia University, Morgantown, WV; d Departments of Neuroscience and Neurosurgery, West Virginia University, Morgantown, WV; e Department of Neuroradiology, West Virginia University, Morgantown, WV; f Department of Neuroscience, West Virginia University, Morgantown, WV; g Department of Neurology, West Virginia University, Morgantown, WV

**Keywords:** Focused ultrasound, Blood-brain barrier, Blood-tumor barrier, Permeability, Drug delivery, Disruption, BBB, Blood-brain barrier, BTB, Blood-tumor barrier, CNS, Central nervous system, ABC, ATP-binding cassette, P-gp, P-glycoprotein, ABCB1, BCRP, Breast cancer resistant protein, ABCG2, MRP, Multi-drug resistant protein, ABCC1, FUS, Focused ultrasound, DCE, Dynamic contrast enhancement, MRI, Magnetic resonance imaging, AUC, Area under the curve, HIFU, High intensity focused ultrasound, LIFU, Low-intensity focused ultrasound, DAMP, Damage associated molecular pattern, TNF, Tumor necrosis factor, HLA, Human Leukocyte antigen, GBM, Glioblastoma, VEGF, Vascular Endothelial Growth Factor

## Abstract

The blood-brain barrier (BBB) is a major anatomical and physiological barrier limiting the passage of drugs into brain. Central nervous system tumors can impair the BBB by changing the tumor microenvironment leading to the formation of a leaky barrier, known as the blood-tumor barrier (BTB). Despite the change in integrity, the BTB remains effective in preventing delivery of chemotherapy into brain tumors. Focused ultrasound is a unique noninvasive technique that can transiently disrupt the BBB and increase accumulation of drugs within targeted areas of the brain. Herein, we summarize the current understanding of different types of targeted ultrasound mediated BBB/BTB disruption techniques. We also discuss influence of the tumor microenvironment on BBB opening, as well as the role of immunological response following disruption. Lastly, we highlight the gaps between evaluation of the parameters governing opening of the BBB/BTB. A deeper understanding of physical opening of the BBB/BTB and the biological effects following disruption can potentially enhance treatment strategies for patients with brain tumors.

## Introduction

Primary and metastatic malignant brain tumors are a leading cause of cancer related deaths in both men and women [1] conferring a 5-year survival rate of approximately 33 percent [1]. Despite extensive preclinical efforts in drug development and therapeutic strategies, treatment remains largely palliative. Lack of effective drug delivery and adequate concentration within brain lesions is a significant limitation in therapeutic efficacy. The blood-brain barrier (BBB) is a protective barrier that limits passage of most therapeutics into brain due to its unique anatomical and physiological properties. The BBB limits paracellular diffusion of therapies from blood to brain secondary to tight junction complexes that seal endothelial cells together. Further, molecules that can cross the endothelial membrane are often extruded back into the vascular compartment by richly expressed efflux transporters like P-glycoprotein (P-gp, ABCB1) and breast cancer resistance protein (BCRP, ABCG2). In case of brain tumorigenesis, an important early step is neo-angiogenesis where the vessels are poorly formed and leakier (blood-tumor barrier; BTB) than the BBB. Despite being leaky, the BTB still inhibits drug permeability to tumors to the degree that they are largely ineffective. Compensatory mechanisms of the BTB like higher efflux, altered active transport mechanisms and refined fluid dynamics effectively reduce drug permeability across the BTB.

An emerging method to improve drug delivery is focused ultrasound to transiently increase BBB/BTB permeability by modulating the integrity of tight endothelial junctions. Although the early experiences are encouraging, current literature lacks consensus in key experimental conditions, limiting our understanding and wide translation of the technique. In this review, we focus on the gaps in current literature to understand drug distribution when BBB/BTB is transiently disrupted by focused ultrasound. We also discuss the role of tumor associated BTB dysfunction and its immunological influence on focused ultrasound mediated physical disruption. Successful disruption of the BBB/BTB by LIFU can potentially overcome the difficulties in drug delivery to brain tumors.

## Blood-brain barrier

The BBB is a unique physiochemical barrier comprised of various cell types, which largely restricts solutes from entering brain from blood. Endothelial cells provide the first barrier between luminal blood flow and abluminal mural cells [2]. The endothelia connect themselves through tight junction protein complexes to form a contiguous barrier limiting paracellular diffusion of most molecules [3]. Adding to this physical barrier, ABC efflux transporters are highly expressed at the luminal and abluminal membrane and can remove a wide variety of lipid-soluble molecules through the numerous transporters including BCRP, P-gp, and Multi-drug Resistance Protein-1 (MRP1, ABCC1) [3,4]. Beyond the initial layer, astrocytes reside on the abluminal side of the BBB and support endothelia through end feet contact, maintaining barrier properties. Embedded in the basement membrane surrounding the capillaries are pericytes, which help regulate cerebral blood flow and contribute to the extracellular matrix [5]. Microglia are resident immune cells of the brain and act as part of the innate immune response. These cells release cytokines in response to a variety of pathological insults that can modify BBB properties [6]. These cells, collectively known as the neurovascular unit, provide a protective barrier, and allow for local and systemic response for the brain in healthy organisms.

## Blood-tumor barrier

The BBB in primary and metastatic brain tumors is anatomically altered and disrupted or “leaky” and referred to as the BTB [7]. To develop a metastatic brain lesion, it is thought that cancerous cells extravasate from their primary site and invade the brain where they colonize and proliferate. After cells have accumulated, the lesion reaches a hypoxic state requiring neo-angiogenesis for further progression [8,9]. During this process, tumor cells secrete VEGF within the hypoxic regions promoting formation and growth of new vessels. The resultant vessels are often abnormal, tortuous, poorly formed, and more permeable compared to the intact BBB [7,8].

Additionally, microvasculature within brain lesions is also disrupted in part by the lack of continuous tight junction proteins creating fenestrations that permit increased solute movement [10]. During lesion formation, the distribution of mural cells (pericyte, astrocytes, and microglia) around the BTB is often irregular, contributing to increased permeability [11].

While altered integrity of the BTB allows increased paracellular transport of molecules, efflux processes at both the BBB and BTB may also be increased by the presence of tumor cells. This may explain mechanistically why many chemotherapeutics fail in the treatment of CNS tumors [10,12]. The major facilitator superfamily domain containing 2A (Mfsd2a) is required for BBB formation and function [13]. In the healthy BBB, Mfsd2a limits transcytosis through modulation of lipid content. Formation and function of caveolae vesicles by brain endothelial cells is prevented by Mfsd2a through control of docosahexaenoic acid transport. However, in the BTB there is a downregulation or complete termination in expression of Mfd2a and other tight junction proteins like ZO-1, claudin-3, claudin-5, and occludin, which have been linked to a higher permeability of the BTB [14,15].

When the BBB is disrupted due to CNS lesions, the change in its integrity is similar to the disrupted BBB observed in CNS inflammatory pathologies, such as multiple sclerosis and neuropsychiatric systemic lupus erythematosus (NPSLE). A clinical study in 2019 showed that the volume transfer constant (K_trans_) for contrast-enhancing lesions was nearly 6.5-fold higher as opposed to non-enhancing lesions [16]. Another study with six NPSLE patients showed significantly higher K_trans_ in the hippocampus than all other regions averaged (*P* < 0.001) compared to control patients [17]. These studies of local and systemic inflammatory disease pathologies are suggestive of the influence of the immune system in disrupting the BBB.

## Heterogeneity of the BTB

Despite presence of leaky vessels in metastatic and primary brain tumors, chemotherapeutics only reach cytotoxic concentrations in less than 10% of brain lesions in preclinical models as well as in patients [7,18]. The reduced accumulation of chemotherapies within brain and tumor lesions can be attributed to a few reasons. First, expression of efflux transporters on the luminal membrane of the BBB/BTB and tumor cells markedly inhibits intracellular accumulation of numerous chemotherapeutics [19].

Secondly, heterogeneity between primary and metastatic brain tumors or different metastatic sites of the same tumor type display varying responses to chemotherapy. Primary and metastatic brain tumors have differential progression, uniquely influencing BTB within the tumor. For example, high grade primary tumors such as glioblastoma have a necrotic core with residual stem cells, a fast-growing central layer and a fully developed envelope which forms the leading edge of the tumor [20]. These layers have different degrees of hypoxia, proliferation rates, and extent of drug permeation.

Third, much of the heterogeneity is because of the unique environment of each tumor region, causing differential release of HIF1, HIF2, IL8, NFκB [21]. Cumulatively, these factors influence unequal drug distribution across the tumor mass resulting in significant challenges in treatment with systemically delivered chemotherapy.

## Formation of a brain metastasis and it's environment

Metastatic cells from distant peripheral sites can disseminate into the vascular system, penetrate the BBB and ultimately invade the brain parenchyma. It is thought the chemokines Mmp3, Mmp9, TNFα, Cxcl12, IL6, IL10, TGFβ promote the metastatic cells ability to infiltrate and proliferate within brain [22]. Further tumor progression and invasion depends on the cell's ability to interact and co-opt the endothelial cells and astrocytes of the BBB.

Different tumor types promote the formation of supporting vasculature that is variable in terms in the number and size of vascular defects. Gliomas have defects or pores that can be 10 times larger than those observed in brain metastases. This significantly alters the total amount of chemotherapy accumulation in the tumor, defining the upper limit of the size of a drug that can be effective in a CNS tumor. Mechanistically this may explain the ability of antibodies to produce an effect in glioblastoma, but why trastuzumab fails as a therapy for brain metastases of breast cancer [23].

Further, immune responses in metastases are unique as compared to those seen in primary gliomas. Brain metastases show decreased concentrations of T-lymphocytes with higher expression of PDL-2 and HLA-1, facilitating the formation of multiple lesions from the circulating cancer cells within brain parenchyma [21].

## Disruption of BBB/ BTB

There is a significant need to design BBB/BTB disrupting techniques to overcome the challenges of delivery of therapeutic agents to target sites within the brain. Current approaches for BBB disruption include intra-carotid injection of a hyperosmotic solution of mannitol, intraparenchymal injection of drug via catheters, radiation-mediated BBB disruption, and use of microbubbles in conjunction with transcranial ultrasound [19]. Among these, microbubble-enhanced focused ultrasound (FUS) is the least invasive, can focally target small brain structures and may have little toxicity on adjacent normal brain cells. Clinically, there appears to be minimal neurotoxicity, inflammation and stroke occurrences associated with the technique [24].

There are numerous studies showing FUS can open the BBB; however, they report variations in BBB opening parameters including power, energy related dose, duration, timing and cycles. Variability within literature limits the ability to provide consensus about optimal parameters needed to deliver a specific therapeutic predictably and repeatedly in targeted regions of the brain (Table 1).

**Table 1:** Preclinical studies of CNS targeted ultrasound.

PMID/ DOI	Aim	Animal/ Cells	Drug/ Tracers	Microbubbles	Time of sonication	Data points (PK, tumor progression, survival)	Major Results/ End point
28848341	Enhance delivery of PTX-LIPO using pulsed LIFU with MBs	Male BALB/c using U87MG cells	long-circulating PTX-LIPO (10mg/kg of body weight)	In-house MBs 0.4-12uM	Using an inhouse transducer MB + drug administered followed by FUS (15sec after drug) 3 treatments over one week	N/A	Higher paclitaxel accumulation following FUS lead to increased survival
**29956460**	Examine use of FUS for enhanced local delivery for glioma	U87-Luc tumors in Female BALB/c mice	IR780- dye PTX-loaded PLGA nanoparticles with 3 mg/kg of PTX	Microbubbles: 1.25 X 10^8 bubbles/kg	For efficacy studies: treatment was administered once every 3 d	N/A	Median survival increased with reduced P-gp signals in sonicated region
27742444	Effect of FUS mediated permeabilization time course of drug accumulation in healthy BBB and tumors	Male Sprague-Dawley rats glioma model (9L rat gliosarcoma)	I.V. injection of microbubbles, MRI contrast agent, DOX (5.67 mg/kg), or Trypan blue.	IV injection of Definity™, 1.2×10^10 microbubbles/mL	60 s sonication immediately after the MB inj. DOX and Trypan blue administered immediately after sonication. Each location was sonicated once	Post FUS, the mean Ktrans value significantly increased in the sonicated brain and tumor	Significantly higher DOX conc at 1 and 24 h in sonicated tumor.
31197598	Anti-tumor activity of carboplatin chemotherapy with and without ultrasonicated BBB	Female Athymic nude-Foxn1nu injected with PDX GBM or U251/U87	Heterotopic: carboplatin ip at 120 mg/kg. Orthotopic: Carboplatin ip 53 or 80 mg/kg/week.	200 μL of Sonovue microbubbles injected iv by retro-orbital route	MB immediately prior to start of ultrasound sonication. Weekly FUS sessions	Whole brain/plasma ratio of carboplatin increased by 4.2-fold	Mice treated with carboplatin + USBBB survived longer with increased survival
30150398	Evaluation of Drug PK in brain metastases after FUS-induced BBB/BTB disruption through mathematical modeling	Human HER2-amplified and estrogen dependent BT474 breast cancer cells in mice	Dox at a concentration of 7 mg/ml over 30 sec Antibody-drug conjugate (T-DM1) (5 mg/kg) i.v.	20 μL/kg, Definity,	MB co-administered with FUS. Chemotherapy administered post FUS. One treatment	T-DM1 and Dox showed varied extravasation due to convective and diffusion related transport, respectively.	Higher Dox and T-DM1 in extravascular regions by transmembrane transport by FUS treatment
30415015	Overcoming efflux of erlotinib using FUS with microbubbles	Adult male Rattus norvegicus Wistar rats	IV injection of 11C-erlotinib (50 MBq/mL) with elacridar and tariquidar	200 µL IV bolus of Sonovue microbubbles	Drugs administered post sonication. One treatment	N/A	FUS-induced BBB disruption did not increase brain uptake of erlotinib due to ABC-mediated efflux
22405901	Examine Dox accumulation and efficacy post HIFU exposures when combined with AP-1-conjugated liposomes	GBM 8401 cells in male NOD-*SCID* mice	Unconjugated Lipo-Dox and conjugated AP-1 Lipo-Dox at 5 mg/kg	SonoVue microbubbles of 2.5um injected at a concentration of 1-5 x 10^8 bubbles/mL.	Lipo-dox and AP-1 Lipo-dox groups were injected prior to HIFU. MB were injected 10s before sonication. Two sessions (D 8 and 12 post tumor implantation)	N/A	Pulsed HIFU enhanced therapy uptake threefold.
23000189	Evaluate delivery of trastuzumab upon FUS and MB exposure to permeabilize BBB/ BTB	HER2/neu-positive human breast cancer cells (BT474) in nude (nu/nu) rats	Trastuzumab (2 mg/kg) through tail vein.	Definity, microbubbles injected at a dose of 10 μl/kg 10 s prior to each sonication.	Trastuzumab was injected immediately after FUS. Six weekly treatments	N/A	The difference in tumor volume between the FUS+trastuzumab group and the three control groups was significant.
**31345243**	Evaluate effects of pulsed FUS with MB on neuroinflammation and vascular damage	Female Sprague Dawley rats	Gadopentetate dimeglumine at 1.66 µL/s	IV infusion of 100 uL of Optison microbubbles	0.3/0.5 MPa peak negative pressure with 10 ms burst length. Groups received either 1,2 or six treatments.	N/A	Weekly FUS with MB resulted in significant pathological changes reflected as sterile inflammation
**29175555**	Assess variability in Dox accumulation in the brain tumor upon FUS-induced BBB disruption	Male NOD-*scid* rats with GMB8401 cells	Bolus Dox injection of 5mg/kg	SonoVue, injected 15 sec before sonication at 300uL/kg	One treatment. Each session was 60 with acoustic power 2.86W and frequency 1Hz	N/A	Dox concentrations derived from tumor-to-contralateral brain ratio in the sonicated tumor tissue was ∼2.35-fold higher than in the non-sonicated tumor tissue
**23029030**	Evaluate the pharmacokinetics of doxorubicin-liposomes upon BBB disruption induced by FUS	Male NOD-scid mice injected with GMB8401 cells	111In-doxorubicin liposomes	I.V injected Sonovue MB	PNP of 0.7 MPa at frequency of 1 Hz. One treatment 5 d after tumor implantation	Tumor-to-contralateral brain ratios from SPECT images were greater after FUS sonication	Lipo-Dox uptake is elevated using FUS and does not confer additional toxicity associated with Dox treatment
**25490097**	Study PKPD and therapeutic efficacy of TMZ when administered with FUS-BBB opening	Pathogen-free male NU/NU mice injected with U87 mice glioma cells.	TMZ orally administered at 50mg/kg	SonoVue SF6-coated microbubbles 4uL/mouse: Diameter 2-5 um	MBs administered before treatment. Animals consumed of TMZ prior to sonication. Two treatments day 1 and 5 of MRI screening.	N/A	FUS group showed 2.7-fold higher TMZ accumulation without change in TMZ plasma dynamics. Degradation of TMZ in tumors was significantly prolonged with better tumor growth suppression at low doses of TMZ
**29471172**	Combination of GNP-Cis conjugates and MRgFUS to focally enhance the delivery of targeted chemotherapeutics to brain tumors.	Human GBM cells U87, U251, T98G, U138 in female NOD SCID Gamma (NSG)	7 nm spherical gold nanoparticles (GNPs) coated with polyacrylic acid (PAA) at Cis (0.5mg/kg) or GNP-UP-Cisplatin (0.5 mg/kg)	0.02 ml/kg of Definity	Prior to sonication, animals were injected with MB. 10-ms bursts, 1-Hz burst repetition frequency, 120 s duration. One treatment		MRgFUS induced increased Gd extravasation and thus increased BBB permeability at the tumor margin of sonicated mice
30534564	Evaluation of pulsed FUS in conjunction with temozolomide for GBM treatment	Wistar rats injected with L9 cell line	Daily intragastric administration of 100 mg/kg temozolomide for 5 d	IV injection of MB containing 8 ul/ml of sulfur hexafluoride	Sonication for 10 mins (1.7/3.3 MHz)	N/A	Lower tumor volume and higher tumor kill in the FUS group (TEM imaging). Western blot showed that claudin-5 levels are reduced in the FUS group.
23527068	Investigate therapeutic use of FUS-induced BBB-disruption to enhance TMZ treatment efficacy.	9L rat glioma cells injected in pathogen-free male Fischer	TMZ was orally administered at 100 mg/kg. OR 50,75,100 mg/kg oral TMZ with or without FUS. EB at 2mg/kg	SonoVue SF6 (0.1 mL/kg bolus mixed with 0.2 mL of saline)	MB prior to FUS. TMZ administered after FUS. Two treatments day 1 and 9	N/A	EB concentration in tumor region increased 2.1-fold with FUS. CSF/Plasma ratio with FUS elevated TMZ concentrations. Tumor volume reduction upon increased local deposition of TMZ
26542745	Analysis of temporary brain activity inhibition through FUS-targeted BBB disruption followed by administration of GABA	Male Sprague Dawley rats	γ-Aminobutyric acid (GABA) administered after sonication	Optison (dose: 200μl/kg) administered before FUS	10ms bursts applied at 1 Hz for 60 s using 690kHz FUS transducer. 1 ms bursts at 9-20 V at 1 Hz. One treatment	N/A	GABA mediated suppression of SSEP lasted 1.5–3.5 h after sonication. Sustained and controlled suppression could be performed by infusing GABA
28288892	Evaluate MRgFUS's ability to increase BPN delivery across the BBB/BTB by monitoring tumor growth and invasiveness	F98 glioma cells implanted in fischer 344 rats and 9L rat glioma cells implanted in Sprague Dawley rats	Nanoparticles were given at 15ug/g body weight. CDDP-BPN was given at a dose of 2.5 mg/kg CDDP.	MB 1E5 MBs/g body weight	MB given pre-FUS Nanoparticles administered post-FUS. Three treatments	N/A	MRgFUS showed homogeneous PS-PEG-BPN delivery (6.4-fold) in 9L tumors and CDDP-BPN showed 30-fold increase in F98 tumors with MRgFUS. Higher MPa of FUS of 0.8 showed a showed a 61% increase in tumor growth inhibition
**16868082**	Feasibility of Herceptin delivery through combination of MR-FUS and MB was examined	10-week-old Swiss-Webster mice weighting 30-35g	Herceptin injected (20 mg/kg) trypan blue (80 mg/kg)	MB-based ultrasound agent Optison (5-8 x 10^8 albumin-coated MB per ml)	Pre-sonication: Herceptin MB;s given during sonication. Post-sonication: trypan blue (80 mg/kg). One treatment	N/A	Post 0.6- or 0.8 MPa sonication, Herceptin in target tissue increased to 1,504 and 3,257 ng/g of tissue.
22818878	Examine impact of FUS mediated therapy on survival in gliomas	9L gliosarcoma cells and male Sprague-Dawley rats	Liposomal doxorubicin (5.67 mg/kg) injected IV	Definity microbubble ultrasonic contrast agent	Pulsed FUS (1.2 MPa 10ms 1 Hz 60-120 s) with concurrent MB. Treatment post-FUS. One treatment	N/A	FUS+DOX showed delayed tumor growth. FUS+DOX group was 26.7% increase in median survival than control by increased penetration.
17437269	Evaluating the use of MRI-FUS for DOX delivery across the BBB. Ultrasound parameters and microbubble concentrations were studied.	Healthy Male Sprague‐Dawley rats.	Dox in pegylated liposomes administered (total DOX dose: 3.0-5.7 mg/kg) **or** i.v injection totaling to 5.7 mg/kg	Optison (5-8 x 10^8 MBs/mL Mean Diameter: 2-4.5 um 0.05-0.5 mL/kg	0.5-2 min durations of pulses at 0.6 W. **Exp 2:** Dox immediately after Optison **Exp 3:** Dox administration after Optison injection. Single or multiple same day sonications	N/A	Dox concentration (819+/-327 ng/g) in brain increased linearly with Optison dose. Consistent BBB opening for 0.6 W or higher. Thalamus, hippocampus, or superior colliculus reproducibly opened with 0.3 W
27742444	Characterization of blood brain barrier permeability following FUS and predictive modeling of doxorubicin delivery	Male Sprague-Dawley rats	DOX administered IV at 5.67 mg/kg	IV injection of Definity microbubbles (10 µL/kg)	10 ms bursts at 1 Hz for 60s, 10- or 120-min intervals DOX and trypan blue given immediately post-sonication. One treatment	Ktrans calculated using MRI contrast agent, DOX concentration over time	Ktrans in single sonication was 2-fold higher while second sonication increased duration of BBB disruption. Linear correlation between DOX concentration and K_trans_ at 30 mins after sonication.
**15588592**	Explore the disruption of the BBB in a frequency range feasible for trans-skull sonications and determine the biological route for material transport into brain tissue.	New Zealand white rabbits (3-4 kg)	N/A	Optison (bolus: 0.05 ml/kg) with MB (mean diameter= 2.0-4.5 x 10^-6^ m) (concentration= 5-8 x 10^8^ /ml)	Optison injected 10s before sonication Pressure amplitudes (0.4-3.1 MPa) or 0.8 and 1.0 MPa. One treatment	BBB disruption evaluated with different sonication pressure amplitudes. Disruption determined by ischemic and apoptotic cells in areas.	BBB disruption at 0.69 MHz causes minimal damage to brain parenchyma cells. 60% of locations had focal contrast enhancement greater than signal in normal brain at 0.4 MPa. By 1.4 MPa, all locations showed BBB disruption.
**20413754**	Feasibility of using FUS to enhance delivery of BCNU to glioblastomas and determine if it increases efficacy	Sprague-Dawley rats with C6 glioma cells (5 x 10^5)	IV injection of single dose of BCNU (13.5 mg/kg)	IV bolus injection of coated microbubbles (Sonovue 2.5 ug/kg)	Single burst-mode (10 ms, 1 Hz repetition frequency) for 30s BCNU post-FUS. One treatment	BCNU concentrations calculated using liquid chromatography	FUS significantly enhanced BCNU penetrance (normal - 340%, tumor - 202%) Increased animal survival and controlled tumor progression
**19546329**	Investigate the effects of targeted disruption of BBB using MRI-guided FUS for methotrexate delivery	Adult male New Zealand White rabbits (2.5-3.5 kg)	IV injection of MTX via ICA	Sonovue (phospholipid shells with sulfur hexa-fluoride)	6W sonication for 6s after sonication and injection of Evans blue (100 mg/kg) One treatment	MTX concentration between sonication group and IV control group	MTX concentration in the sonicated group (7.412 ug/g) was significantly higher than IV and ICA groups (0.544, 1.984 ug/g)
**26566207**	Effect of FUS-BBB opening on the intracerebral concentration of TMZ and irinotecan	Healthy male New Zealand white rabbits	Irinotecan (CPT-11) I.V (6 mg/kg) and TMZ at a mean dose of 4.7 mg/kg	Sonovue contrast agent	0.6 MPa, 1 Hz repetition frequency, 23.2 ms. One treatment	TMZ and CPT-11 quantification in plasma and brain via liquid chromatography	Mean intracerebral tissue-to-plasma concentration ratio post-sonication increased to 21% for TMZ and 178% for CPT-11
25784614	Effect of FUS to temporally open the BBB and evaluate synergistic effect from concurrent interleukin-12 to trigger local immune responses	Male Sprague-Dawley rats (200-225 g) and C6 glioma cells (1x10^5 cells/mL	IL-12 injected intraperitoneal (0.3 ug/kg/day) for five d. Evans blue tracer used.	0.1 mL/kg MBs followed with flushing of 0.2 mL heparin.	0.36-0.7 MPa single sonication burst mode: burst length 100ms, 1 Hz, exposure time 90s. Three treatments d 11,13 and 15 post-sonication	N/A	Exposure power level 5W showed successful BBB opening while 20W exposure showed BBB opened regions spreading toward a wider area.
27496633	Evaluating the treatment effect of FUS-induced BBB disruption in combination with trastuzumab and pertuzumab	Male nude rats injected with MDA-MB-361 cells (2 x 10^6^)	Transtuzumab and pertuzumab at 4 mg/kg for week 1 and 2 mg/kg for the weeks after	100 ul/kg of Optison contrast agent was injected	Drug injected pre-sonication. 10 ms burst, 1 Hz repetition frequency, 60s duration. 0.46/0.62 MPa. Six sonication sessions	Tumor growth rates between treatment groups	Only 4/10 animals responded to treatment and exhibited a lower tumor growth rate (0.01 cubic mm/day compared to 0.043)
**27192459**	Demonstrate that MR-guided FUS can enhance delivery of bevacizumab into brain for treatment of GBM	Male NU/NU mice injected with U87 glioma cells (5 x 10^5^)	Injection of radiolabeled (gallium 68) bevacizumab for PET imaging post-sonication	10 ul Sonovue sulfur hexafluoride filled MBs	Burst-tone mode ultrasound (10 ms), pulse repetition frequency = 1 Hz, exposure time = 60s. 5 treatments	Bevacizumab penetration into CNS, glioma progression, median survival time	Bevacizumab penetration increased by 5.7-56.7-fold in the FUS model. 135% median survival time in treatment group
23640533	Increasing uptake of boronopheny-lalanine-fructose complex (BPA-f) by using MR-guided FUS	Male Fisher 344 rats implanted with 9L gliosarcoma cells (2.5 x 10^5^)	BPA-f (250 mg/kg). 25% delivered as initial bolus and remainder delivered over a 2hr infusion	0.02 ml/kg Definity bolus via tail vein catheter	10 ms pulse repetition frequency = 1 Hz, duration = 120s. One treatment	Mean tumor concentration of BPA-f and tumor-brain ratio, boron uptake in infiltrating clusters	Ultrasound increased the accumulation of BPA-f in tumor and infiltrating cells (6.7 vs 4.1 tumor-brain ratio)
24936788	Evaluate the concentration-time prolife of boron in brain tumors with FUS exposure in comparison to non-sonicated brain tumors	Male Fisher 344 rats injected with 1 x 10^5^ F98 rat glioma cells	Intravenous bolus injection of BPA-fr (500 mg/kg)	Sonovue ultrasound contrast agent was injected into the femoral vein (300 uL/kg)	Sonication of 60s with burst length of 50 ms, repetition frequency = 1 Hz, sonication. One treatment	Unbound BPA in tumor ECF and plasma, dialysate and perfusate by microdialysis	Mean peak concentration of BPA-f in the glioma was 3.6 times greater in the FUS treatment group, AUC of concentration-time curve is 2.1 times greater
10.1126/sciadv.aay1344	Evaluate FUS+MB-mediated BTB/BBB with BPNs for targeted tumor transfection and its effect on tumor interstitial fluid flow and BPN transport	Athymic nude mice (U87mCherry glioma and B16Flova	Iv 0.05mL of gadolinium contrast agent with Luc-BPNs (1ug/g body weight)	albumin-shelled MBs ( 1x10^5/ g body weight and	FUS applied using 0.45 or 0.55 MPa in 10-ms pulses with a 2-s pulsing interval for 2 min. One treatment session	N/A	FUS-mediated BTB/BB opening augmented interstitial tumor flow 2-fold which plays a major role in enhancing BPN dispersion (>100%) through tumor tissue.
**32472017**	Reliability of FUS mediated disruption of BBB for irinotecan delivery	Male and female Sprague Dawley or Fischer rats	Irinotecan 10-20mg/kg	Definity MB 10µl/kg	5ms bursts or 1.1Hz. Power 0.16-0.39W, 68-165kPA. Three weekly sessions	N/A	Irinotecan post BBB disruption increased, but <50% samples showed SN-38. No effect on tumor growth/ survival.
**31999201**	Temporal effects of FUS post radiotherapy in brain tumors	C57B6 mice without tumors	6GyX5 administered before FUS as chronic and acute exposures	N/A	0.72MPa, 5Hz for 30secs. One treatment	Generic kinetic model used to determine permeability (K_trans_) of opened region	Non-significant increase in Ktrans, Gd enhancement and higher vascular density in acute exposure. Differences not seen in chronic exposure
**32534883**	Safety and efficiency of non-focused US with lipid MBs	six-week-old male mice	fluorescence-labeled dextran (3, 70, or 2000 kDa, 2 mg/mouse) or DiO-labeled liposomes and EB 5mg/mouse	Lipid MBs (50 nmol of lipids/mouse)	1, 3 ,10MHz, duty 50%, PRF; exposure time; 3 min). (frequency; 0.1, 0.5, 1.0 or 2.0 W/cm^2.^ Single treatment	N/A	Unfocused US+MB induced reversible BBB opening and 2000kDa molecule delivery

## Ultrasound applications within brain

In the following sections, we will discuss the multiple forms of ultrasound that have been used for therapy and/or augmentation of therapy for tumors within the CNS. We will also highlight the physiologic and immunological response to the opening as well as the ability of the technique to improve drug distribution and effect in tumors. In general, ultrasound is specifically targeted to areas within the CNS using intra-treatment magnetic resonance imaging (MRI). Multiple wavelengths or “intensity” of the ultrasound wave, with and without vascular microbubbles are used for a variety of applications including ablation of small brain regions or opening of the BBB or BTB.

## High intensity focused ultrasound

High intensity focused ultrasound (HIFU) utilizes a stereotactic device to distribute high intensity energy (100–10,000 W/cm^2^) through the skull. This produces spatial ablation at target tumor sites by increasing the temperature to approximately 55^0^C. Cell death is induced by the thermal energy deposited, frictional vibration between cells or non-thermal pulsed changes in peak rarefaction pressure amplitude [25]. High intensity focused ultrasound is currently FDA approved for essential tremor and tremor dominant Parkinson's disease to create an ablation in thalamus to modulate the neural circuitry of the tremor [26,27].

The current HIFU system is limited in terms of brain tissue volume that can be ablated, which is an important consideration for tumor ablation [26]. Interstitial HIFU is an alternative to the traditional technique to circumvent this limitation. Here, single or multi-elemental catheters with cylindrical cooling elements deliver high ultrasound energy within the parenchyma of intracranial neoplasms. In a swine model this method was highly effective for tumor ablation [28]. A unique advantage of interstitial HIFU is that it can be used for theranostic purposes using a cannula and catheter for simultaneous biopsy and treatment. Further, it limits issues related to near-field heating or patient motion during longer therapy sessions by providing the ability to tailor heating patterns that conform to the tumor allowing precision in treatment margins [28].

While HIFU is clinically used for ablation, evidence from pre-clinical models suggests there are secondary immunomodulatory effects of the tumor microenvironment post tumor ablation [25]. The anti-tumor immunological response possibly arises from activation of the dendritic cells along with an increase in the CD4+, CD3+ as well as the ratio of CD4+/CD8+ cells in the blood [25,29,30]. Currently, the combination of HIFU with PDL-1 antibody blockade is being investigated clinically in solid tumors outside the CNS (NCT04116320). The effect of HIFU combined with immunotherapy in CNS tumors remains to be evaluated.

## Low intensity focused ultrasound

Targeted disruption of the BBB or BTB can be achieved using low intensity focused ultrasound (LIFU) at lower frequencies. In this technique, ultrasound waves are co-exposed with intravenously administered gas-filled bubbles that are composed of perfluorocarbon encapsulated in phospholipid formulations. These microbubbles undergo stable oscillations to produce a transient vessel permeabilization [31]. Due to the mechanical effect, and its non-invasive nature, LIFU may eventually substitute other procedures such as transcranial magnetic stimulation or deep brain stimulation which potentially risk strong immune response or infection [32]. LIFU combined with advanced imaging modalities such as dynamic contrast enhanced magnetic resonance imaging (DCE-MRI) has allowed additional insight and therapeutic applications to different pathologies including primary and metastatic brain tumors (Table 2).

**Table 2:** Current HIFU and LIFU CNS tumor clinical trials.

NCT Number:	Study Start:	Study Title	Type of CNS Tumor	Outcome Measures	Status/ Results
NCT01698437	Tuesday, February 1, 2011	Magnetic Resonance (MR) Guided Focused Ultrasound in the Treatment of Brain Tumors	(Malignant or recurrent glioma or supratentorial brain metastasis	Safety of patients associated with lesion size	Completed/ Not reported
NCT01473485	Friday, April 1, 2011	ExAblate (Magnetic Resonance-guided Focused Ultrasound Surgery) Treatment of Brain Tumors	Recurrent or progressive glioma or metastatic brain tumors	Evaluation of device safety	Active, not recruiting
NCT00147056	Wednesday, August 1, 2012	MRI-Guided Focused Ultrasound Feasibility Study for Brain Tumors	Newly diagnosed or recurrent metastatic tumors	Adverse events classified as serious and non-serious events post MgFUS in brain tumors	Active, not recruiting
NCT02343991	Wednesday, October 1, 2014	Blood-Brain Barrier Disruption Using Transcranial MRI-Guided Focused Ultrasound	Gliomas	Adverse events related to device and procedure parameters which are classified based on number and severity.	Active, not recruiting/ First few procedures well tolerated with peritumoral and tumoral contrast enhancement of about 15-50 percent
NCT03028246	Tuesday, February 28, 2017	A Feasibility Safety Study of Benign Centrally-Located Intracranial Tumors in Pediatric and Young Adult Subjects	Benign Centrally-Located Intracranial Tumors	Adverse events following treatment and tolerability based on tumor volume and general physical exams.	Recruiting
NCT03626896	Friday, August 17, 2018	Safety of BBB Disruption Using NaviFUS System in Recurrent Glioblastoma Multiforme (GBM) Patients	Recurrent GBM	Extent of BBB disruption by NaviFUS with monitoring of dose tolerability and adverse events classified in number and severity.	Completed, No results posted
NCT03712293	Tuesday, August 28, 2018	ExAblate Blood-Brain Barrier Disruption for Glioblastoma in Patients Undergoing Standard Chemotherapy	Glioblastoma Multiforme	Safety profile of BBB opening through adverse event monitoring	Recruiting/ Repetitive focused ultrasound on same spot was well tolerated with no delayed complications
NCT03616860	Tuesday, October 16, 2018	Assessment of Safety and Feasibility of ExAblate Blood-Brain Barrier (BBB) Disruption for Treatment of Glioma	Grade IV, Malignant glioma (GBM)	Feasibility, effectiveness and repeatability of device and procedure for tumor therapy through MR imaging to observe adverse events	Recruiting
NCT03551249	Tuesday, March 26, 2019	Assessment of Safety and Feasibility of ExAblate Blood-Brain Barrier (BBB) Disruption	Grade IV glioma (GBM)	Feasibility, effectiveness and repeatability of device and procedure for tumor therapy through MR imaging to observe adverse events	Recruiting
NCT03714243	Wednesday, September 18, 2019	Blood Brain Barrier Disruption (BBBD) Using MRgFUS in the Treatment of Her2-positive Breast Cancer Brain Metastases	Her2-positive brain metastases of Breast Cancer Brain Metastases	Feasibility of BBB disruption without adverse events	Recruiting
NCT04446416	Wednesday, July 1, 2020	Efficacy and Safety of NaviFUS System add-on Bevacizumab (BEV) in Recurrent GBM Patients	Recurrent Glioblastoma	Evaluation of treatment outcome by monitoring of tumor shrinkage, progression free survival at 6 months and adverse events	Not yet recruiting
NCT04440358	Saturday, August 1, 2020	Exablate Blood-Brain Barrier Disruption with Carboplatin for the Treatment of rGBM	Recurrent Glioblastoma	Number of Adverse Events detected through MR imaging contrast enhancement	Not yet recruiting
NCT04417088	Tuesday, September 1, 2020	Exablate Blood-Brain Barrier Disruption for the Treatment of rGBM in Subjects Undergoing Carboplatin Monotherapy	Recurrent Glioblastoma	Number of Adverse Events related to carboplatin therapy. Detected through MR imaging contrast enhancement	Not yet recruiting

## Mechanism of LIFU mediated BBB disruption

Disruption of the BBB at the vascular endothelia has been described by multiple mechanisms of interaction of LIFU and microbubbles, but none have been confirmed. A primary hypothesis suggests ultrasound waves force microbubbles to oscillate, resulting in increased vessel pressure, tight junction expansion and increased membrane permeability. A second hypothesis not exclusive of the first, suggests microbubble oscillation can activate and increase expression of cellular receptors or transcytoplasmic shuttling vesicles. This potentially increases transcellular permeability through a caveolin dependent mechanism where transport across arterioles and endothelia is increased by vesicular fusion and formation of transcytotic channels [33,34].

In both cases, 2 types of microbubble oscillations have been described. Under the influence of ultrasound, microbubbles can produce stable (noninertial), and inertial oscillations, which are termed as cavitations. Effect of the cavitations on the BBB can be defined by a mechanical index; the negative acoustic pressure over the square root of the frequency, or the cavitation index; the negative acoustic pressure over frequency [35]. The mechanical index defines biological effects produced mechanically by sonication, while cavitation index measures the scale of stable cavitation involved in FUS induced opening [35]. At low mechanical indices, microbubbles oscillate in a linear and uniform way and produce harmonic or sub-harmonic emissions. These oscillations are equivalent to the mechanical index applied (Fig. 2) [36].

$$\text{CI}=\frac{P\left(\text{MPa}\right)}{f\;\left(\text{MHz}\right)}$$

**Fig 1:**
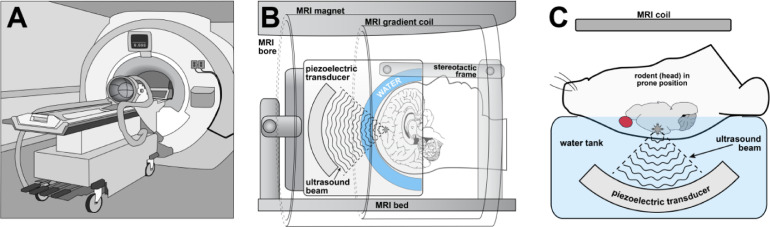
Application of the transcranial MR-guided LIFU in pre-clinical and clinical settings. (A) The phase array piezoceramic helmet-shaped transducer is placed on the positioning table before mounting in the MRI scanner. (B) Pre-treatment MR images and MR compatible stereotactic frames allow precise positioning of focal points in the transducer to deliver ultrasonic beams in humans. (C) Animals are placed in a holder so that their head lays in a supine position with the skull touching the degassed water inside the transducer for accurate delivery of ultrasonic beams.

**Fig 2:**
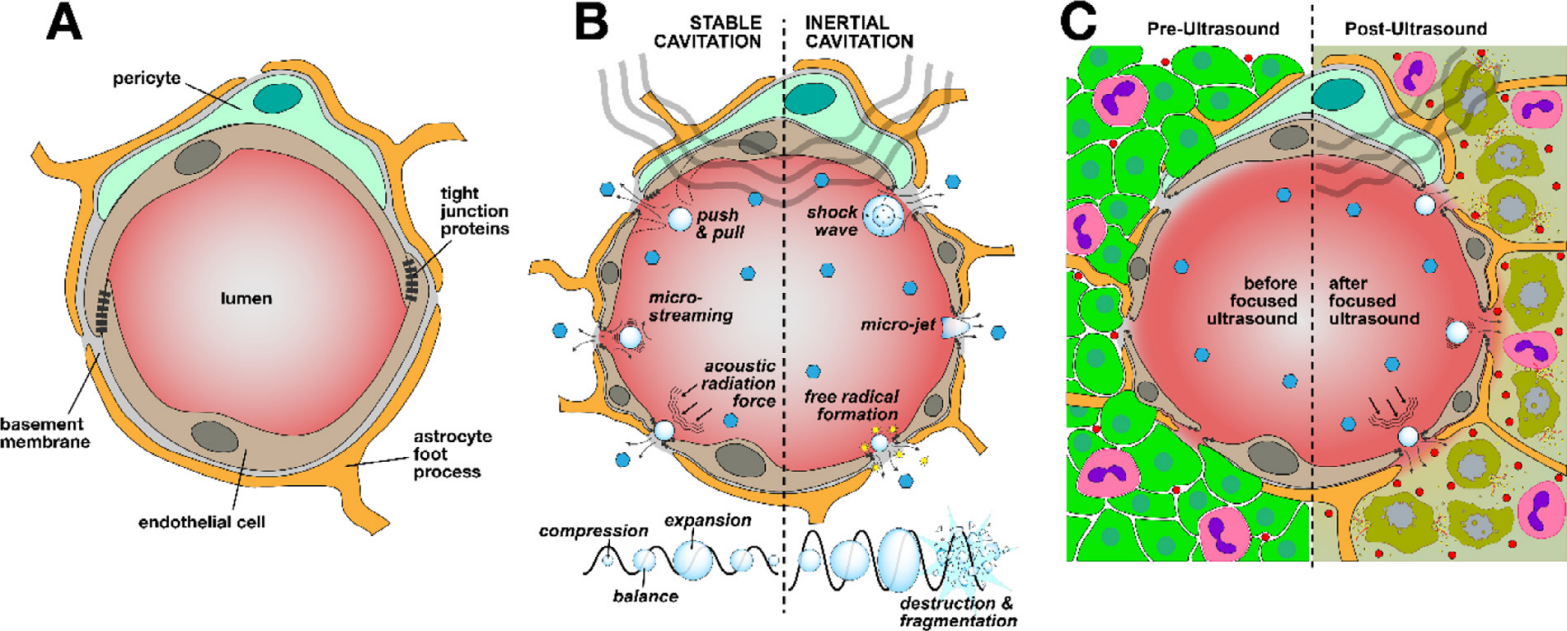
Exposure to LIFU alters BBB/BTB permeability through transient disruption of tight junction proteins. (A) Brain endothelial cells in a healthy BBB inhibit paracellular transport through presence of tight junctions formed by the neurovascular unit. (B) Harmonic and sub-harmonic oscillations of the microbubbles under ultrasonic exposures cause loosening of the tight junction proteins through stable or inertial cavitations. (C) In brain tumors LIFU causes increased delivery of chemotherapeutics to tumors causing additional tumor cell apoptosis.

Stable cavitations are produced as a result of an equal amount of gas efflux and influx within the microbubbles causing their rhythmic expansion and contraction. When microbubble expansion occurs, there is a stretching of the vessel which may open cell-cell junctions transiently [37]. Oscillation of microbubbles produces micro-streams which induces shear stress on vascular endothelia resulting in increased rate of endocytosis (Fig. 2B) [38].

Stable cavitation can also cause acoustic radiation forces, where microbubbles are pushed towards endothelia resulting in a “kneading” or pounding effect leading to increased passive permeability [37].

Not all cavitation is well contained within the microbubbles. At times, higher mechanical indices will cause microbubbles to oscillate rapidly eventually resulting in bubble fragmentation. Bubble collapse causes microjet formation and small shock waves (Fig. 2B) directed at the endothelia, resulting in increased permeability [37]. Lastly, a more tangential or longer acting mechanism assumes that oscillating microbubbles increase local endothelial temperatures, which increases permeability through protein expression changes and not direct mechanical interactions [39].

While the modulatory effects of LIFU on endothelial cells is currently being investigated, effects on other cells of the neurovascular unit remains largely unknown. Preliminary studies indicate that mechanical disruption of the BBB leads to transient activation of microglia and astrocytes mediated by inflammatory mechanisms that can last upto 24 h postdisruption [40]. While increased clearance through astrocytic and microglial phagocytosis is expected post-LIFU, change in other homeostatic roles is yet to be investigated. For example: it is known that astrocytes may play an important role in cerebrospinal fluid clearance through AQP4 channels [41]. However, the effect of LIFU on these channels is unknown. There is also evidence of reduction in arterial blood flow post LIFU potentially mediated by neurovascular coupling, vasospasms, disrupted neurovascular signaling and suppression of neuronal response [41]. Change in clearance of CNS active drugs through these mechanisms post-LIFU needs to be elucidated.

## Immune effects in BBB ultrasound LIFU disruption

**Fig 3:**
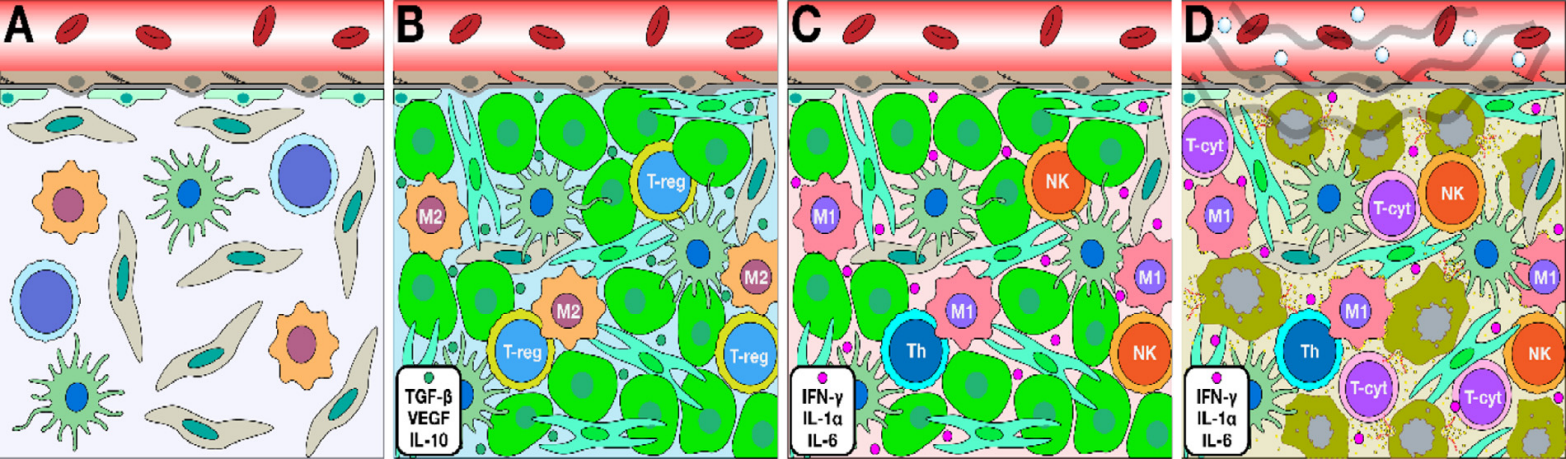
Immunological milieu in brain alters in response to tumor and LIFU microenvironment. (A) Healthy brain is equipped with astrocytes, dendritic cells and the microglia which are the resident innate immunological cells that carry out phagocytosis of residual debris in the brain. (B) Pro tumor microenvironment demonstrated by the presence of tumor-associated macrophages (TAMS) with an M2 phenotype, regulatory T-cells (T-Reg) that suppress cytotoxic CD8+ cells and myeloid suppressor cells. (C) Anti-tumor microenvironment shows suppression of the tumor cells through release and modulation of TH1 CD8+ and CD4+ cells that further promote release of IL-1, TNF-alpha, and interferon gamma. (D): LIFU has been suspected to alter the immunological milieu to promote the anti-tumor immunological microenvironment by increasing maturation of dendritic cells, release of chemokines and tropic factors, as well as promotion of T-cells to the tumor endothelial to increase tumor cell death.

The brain has been considered an immune-privileged site since the early 1900s through studies demonstrating tissue transplants into the brain parenchyma could occur without host rejection, despite a differential immunological response in peripheral implants. It is widely accepted there is immunosurveillance within the brain, which can elicit strong immune responses [42]. Unfortunately, immunotherapies have largely been ineffective in treating CNS tumors due to poor penetration of therapeutics across the BBB and subsequently lack of activation of the CNS immune system. Further, often in high grade CNS tumors, such as glioblastoma multiforme (GBM) there is decreased effector T cells and increased T regulatory cells which shifts the tumor microenvironment to immunosuppressive and promotes tumor growth (Fig. 3) [30].

There is a gap in understanding of the exact mechanism behind local and systemic post ultrasound immunomodulation. It is hypothesized that damage-associated molecular patterns (DAMPs) are produced by endothelial cells in response to the microbubbles cavitation to activate and recruit proinflammatory immune cells [43]. Changes in the local and systemic immunological environment significantly impacts BBB permeability [44]. Using LIFU to mechanically open the BBB results in influx of inflammatory cells and markers into the brain, potentiating further BBB disruption and promoting immune cell activation [42, 43, 44]. Ultrasound and microbubble mediated cavitations at tight-junctions induce changes in the expression of integral proteins, Ca^2+^ influx and transient detachment of endothelia from the extracellular matrix [43,44]. A recent study by Hynynen et al. provided evidence of peripheral immune cell recruitment at the BBB immediately after LIFU mediated sonication [45]. Interaction between vascular endothelia and oscillating microbubbles causes an immediate but transient response by circulating neutrophils [41]. Further, the initial infiltration leads to an acute inflammatory cascade by release of chemokines and recruitment of more immune cells such as monocytes and phagocytes [45].

Initiation of immune responses due to physical changes within the vasculature can increase permeability across the barrier. Microbubble mediated disruption has also been implicated in slowing down blood perfusion by vasoconstricting the larger vessels, which may also contribute to increased BBB permeability [46]. The vasoconstricted vessels may mediate hypoxic stress responses through increased levels of heat shock protein 40, VEGF, erythropoietin, IL1α, IL1β and TNFα in the parenchyma [43] **(**Fig. 3).

Recent studies show BBB opening using LIFU induces sterile inflammation for a minimum of 24 h [43]. Although not completely understood, the underlying mechanism may be through immediate triggered release of damage associated molecular patterns (DAMPs) from endothelia [43]. DAMPs like HMGB1 may induce sterile inflammation via the NFκB pathway and can be correlated with increased BBB permeability [47]. Elevation in levels of pro-inflammatory and tropic factors is also seen concurrently with sterile inflammation. Increased innate immunity responses up to 6 d post sonication is seen through infiltration and the continued presence of CD68+ macrophages. In a preclinical rat glioma model, LIFU increased intra and inter tumoral cytotoxic T cell populations [48]. When the T-cell activating cytokine IL-12 was administered with LIFU, an increase in cytotoxic T cell to T regulatory cell ratio was observed and appeared to correlate with an increase in overall survival [48]. Immunomodulation with LIFU may also cause antigen release into the bloodstream from the tumors in the CNS, resulting in an induction of a pro-inflammatory environment [49]. The antigens released are captured by peripheral antigen presenting cells (APCs) leading to T-cell activation. Primed T-cells then infiltrate the tumor by adhesion to tumor endothelia which results in apoptosis [49].

Similar immunological changes are observed with other techniques of BBB disruption like radiotherapy where a 10Gy dose in combination with chemotherapy/immune checkpoint inhibitors upregulated proinflammatory markers like CCL2, CCL11, and IL-6 [61]. The aggregate data suggest there are multiple underlying mechanisms by which the immunological milieu regulates BBB disruption. Further, studies are needed to evaluate balance between pro-inflammatory and anti-inflammatory responses.

## Unfocused ultrasound

In contrast to application of LIFU for targeted disruption of the BBB, unfocused ultrasound with microbubble cavitation induces a broad opening of the BBB, which may be advantageous to deliver therapeutics for diffuse pathology. To accomplish this, a transducer is implanted within the skull, which provides a controlled distribution of the ultrasonic energy coupled with lower attenuation by the skull [50]. Device implantation allows for long-term, repetitive disruption without the need for MRI guidance. Similar to the studies described above, pre-clinical unfocused ultrasound has shown BBB disruption as evidenced by a 4-fold increase in cortical Evans blue concentration in the sonicated hemispheres of rabbits as well as a significant increase in MRI Gd enhancement [51]. Further studies have demonstrated increased tissue-plasma drug concentration ratio of temozolomide and irinotecan in between control and sonicated hemispheres. Clinical utility was demonstrated in a recent study where BBB disruption at higher acoustic pressures (1.1 MPa) resulted in cortical Gd enhancement without detectable adverse effects [52]. Despite its potential, unfocused ultrasound is primarily used for diagnostic and imaging purposes due to lack of available data regarding its use to target deep seated structures in the brain [53].

## Factors influencing efficacy of LIFU mediated BBB disruption

Within this section, we will discuss the differences in instrumentation, the set-up and or parameters that influence the ability to produce BBB disruption.

The type of transducer used to emit the ultrasound directly affects the wave's propagation to brain and can profoundly influence efficacy of LIFU BBB disruption. One of the major obstacles in achieving targeted LIFU penetration is the skull's high impedance [37]. To overcome this, a geometrically archetypal transducer is required to prevent propagated wave distortion due to bone irregularity. Similarly, phase-array transducers reduce skull attenuation and undesirable heating due to improved focal volume dimensions which concentrate ultrasonic energy to focal regions [54]. Limited repeated application of multi-array transducers to superficial and deep-seated tumor lesions also gave rise to implanted cranial transducers like SonoCloud [54]. Efficacy of SonoCloud was noted in a recurrent glioblastoma with ultrasonication dose escalation prior to carboplatin administration [52]. This study revealed pressures up to 1.1 megapascal were well tolerated using pulsed ultrasound with an implanted transducer [52,55]. Other preclinical studies using small animals and primates also suggested higher safety margins and increased drug distribution with the same transducer [56,57]. A separate study suggested 8% yttria-stabilized-zirconia polycrystalline ceramics (8YSZ) as a biocompatible alternative to implantable transducers with an 81% maximum transmission efficiency [58].

Another factor affecting experimental outcome is the type, formulation and concentration of intravenously administered microbubbles. Multiple data sets have compared the effect of different commercially available, FDA approved microbubbles like SonoVue®, Optison and Definity® which differ in diameter, concentration, sizes, composition and pharmacokinetic parameters [59, 60, 61]. While some pre-clinical studies have indicated that no significant differences in BBB permeability occur due to microbubble concentrations; others suggest microbubble concentration and dose have differential delivered drug concentrations up to 6-fold and continue to affect BBB permeability up to 6 h post administration [62,63]. McDannold et al., demonstrated similar acoustic thresholds yield different extent of BBB opening using Optison and Definity depending on polymers and lipids composed within the microbubble shell [63,64].

Other factors influencing ultrasound mediated BBB disruption include pressure amplitude, frequency and power. Pressure amplitude affects resonance of the microbubbles; and appears to directly positively correlate to increasing BBB permeability. It should be noted that a pressure amplitude above of 0.47 MPa at a 300 second exposure time results in irreversible damage to brain endothelia [65]. Similarly, lower ultrasound frequency results in lower impedance through the skull which allows an increased BBB permeability [66,67]. However, removing the skull via craniotomy results in an opposite relationship: higher frequencies causes greater BBB permeability [63]. Lastly, increases in pulse repetition frequency from 0.1-20 Hz and pulse length between 0.1 and 20 ms produces enhanced BBB permeation [37,63,68].

## Drug delivery using LIFU mediated BBB disruption

The extent of BBB opening is often correlated to the size and extent of drug permeation. In a HER-2 positive brain metastases of breast cancer preclinical model a seven-fold higher accumulation for the small molecule (doxorubicin) and a 5-fold increased accumulation of ADC (ado-trastuzumab emtansine T-DM1) was observed post LIFU [69]. Data from the work suggested small molecule penetration may be related to convective transport, higher diffusion and hydraulic conductivity. While increased penetration was observed for the larger T-DM1 molecule, it was only seen immediately following FUS. Another preclinical study showed 2.35-fold increase in doxorubicin accumulation in FUS treated tumor fractions of a GBM model, with a 3.3-fold higher AUC analyzed by intracerebral microdialysis [70].

BBB disruption with LIFU was able to improve paclitaxel loaded liposomes and PLGA nanoparticles distribution in a nude mouse model of glioblastoma with higher accumulation for the liposomes [71]. Increased FUS mediated permeability has been reported for other small molecules such as doxorubicin, irinotecan, cisplatin, temozolomide, cytarabine as well as antibodies such as trastuzumab, pertuzumab, bevacizumab, and their nanoparticulate formulations (Table 1).

## Clinical impact of FUS mediated BBB/BTB disruption

While many pre-clinical studies have individually demonstrated opening of the BBB/ BTB, very few trials have provided preliminary proof of clinical implementation of this technology. Clinical application of FUS is currently being tested in a few pathologies including primary tumors, Alzheimer's disease, essential tremor, Parkinson's disease and amyotrophic lateral sclerosis [72, 73, 74]. One of first clinical reports of HIFU used the ExAblate 3000 which could reduce acoustic backscatter and beam dispersion due to skull thickness variability [75]. This technology was coupled to an MRI to perform a guided surrogate surgical resection. However, there was insufficient power to generate the higher temperatures needed to produce thermal ablation. A later 2014 study overcame power related issues, but was limited by acoustic impedance through bone attenuation, time required for ablation and effects on healthy tissues [76]. Recently, many studies have attained pre-clinical success in achieving controlled ablation of the tumoral tissue by modification of acoustic specifications [77,78]. For example, when the transducer parameters including power, duty cycle and frequency are controlled a functional thermal dose can be obtained [77]. A recent study evaluated BTB disruption using FUS with adjuvant temozolomide and showed accurate and safe opening in patients for six sonication cycles at the same targeted sites [74]. Another recent investigation reported feasibility of BBB opening within peri-enhancing regions of the brain in six patients with recurrent GBM, using the NaviFUS system with concomitant Sonovue microbubbles [79]. This analysis suggests a dose-dependent BBB permeability effect of FUS, based on DCE kinetic parameter analysis (K_trans_ and V_e_) [79].

A distinct advantage of FUS is the precise, and reversible on demand BBB opening in the region of interest including the deep brain targets besides being noninvasive. Our group demonstrated safe and reversible opening of BBB in the complex and deep-seated structures of hippocampus and entorhinal cortex in patients with Alzheimer's disease [80]. The BBB opening was immediate and sizeable consisting of about 29% of hippocampus and the BBB closed within 24-h post sonication [80]. As evident from the basic science studies suggesting immunomodulation, MRI investigation revealed perivenous enhancement during acute BBB opening which persisted even after BBB closure, suggesting a downstream immunological response blood-meningeal barrier in patients with Alzheimer's disease [73].

In the case of primary malignant tumors there have been reports of successful BBB opening and enhanced delivery of chemotherapeutics [55,81]. While initial clinical implementation of FUS has demonstrated safe, reproducible, and repeatable opening of the BBB, the long-term effects of this modality need to be delineated. Available clinical data encompasses various FUS devices and microbubble contrast agent as well as heterogeneous procedural and technical parameters. These preliminary studies have demonstrated the need to understand secondary effects accompanying BBB sonication under fixed procedural parameters. Despite these challenges, it is expected that the use of FUS to deliver therapeutics across the BBB for CNS malignancies and neurological conditions will increase in the coming years.

## Conclusion

Magnetic resonance guided focused ultrasound is a non-invasive technique increasingly explored to treat various stages of cancers. An MRI provides detailed anatomical images with the capability of precise targeting a tumor region within the body [82]. The combination therapy of LIFU and MRI facilitates the localization, targeting, and real-time monitoring while simultaneously minimizing collateral damage to surrounding normal tissues [82]. The method of disrupting BBB by using LIFU to oscillate intravenously injected microbubbles may improve the distribution and efficacy of therapeutics to brain tumor sites. Additionally, the safety and reproducibility of this technique has been demonstrated by a few pre-clinical and clinical studies [55,83]. Nevertheless, there are limitations, which includes identifying optimal ultrasound parameters. Suboptimal parameters can induce hemorrhages, erythrocyte extravasation, and edema formation, while weak parameters may not have therapeutic effect [37]. Further work still needs to be done to understand the correlation between microbubble size (or concentration) and duration of BBB openings [37]. Other limitations, such as a lack of portability, long duration time for the treatment, an inability to monitor true acoustic cavitation on focused ultrasound therapies and selection of correct statistical methods to normalize data analysis gathered by multiple parameters needs to be overcome [83]. Despite these limitations, MRI guided focused ultrasound provides a novel way to increase drug distribution to brain through a reversible and on-demand opening of BBB. Further translational clinical studies are needed to explore the potential of this technology.

## Declaration of competing interest

The authors declare that they have no known competing financial interests or personal relationships that could have appeared to influence the work reported in this paper.
